# Does environmental policy affect scaling laws between population and pollution? Evidence from American metropolitan areas

**DOI:** 10.1371/journal.pone.0181407

**Published:** 2017-08-09

**Authors:** Nicholas Z. Muller, Akshaya Jha

**Affiliations:** 1 Department of Engineering, Public Policy, and Management and Tepper School of Business, Carnegie Mellon University, Pittsburgh, PA, United States of America; 2 Heinz College of Information Systems and Public Policy, Carnegie Mellon University, Pittsburgh, PA, United States of America; Peking University, CHINA

## Abstract

Modern cities are engines of production, innovation, and growth. However, urbanization also increases both local and global pollution from household consumption and firms’ production. Do emissions change proportionately to city size or does pollution tend to outpace or lag urbanization? Do emissions scale differently with population versus economic growth or are emissions, population, and economic growth inextricably linked? How are the scaling relationships between emissions, population, and economic growth affected by environmental regulation? This paper examines the link between urbanization, economic growth and pollution using data from Metropolitan Statistical Areas (MSAs) in the United States between 1999 and 2011. We find that the emissions of local air pollution in these MSAs scale according to a ¾ power law with both population size and gross domestic product (GDP). However, the *monetary damages* from these local emissions scale linearly with both population and GDP. Counties that have previously been out of attainment with the local air quality standards set by the Clean Air Act show an entirely different relationship: local emissions scale according to the square root of population, while the monetary damages from local air pollution follow a 2/3^rds^ power law with population. Counties out of attainment are subject to more stringent emission controls; we argue based on this that enforcement of the Clean Air Act induces sublinear scaling between emissions, damages, and city size. In contrast, we find that metropolitan GDP scales super-linearly with population in all MSAs regardless of attainment status. Summarizing, our findings suggest that environmental policy limits the adverse effects of urbanization without interfering with the productivity benefits that manifest in cities.

## Introduction

Modern cities are engines of production, innovation, and growth [1,2]. People gravitate to cities to improve their quality of life while firms locate in cities to boost productivity. The power of these forces is evident: the global population is urbanizing. However, increased urbanization potentially has environmental costs. This paper explores the trade-off between the economic benefits versus environmental costs of urbanization, asking the following questions: (1) Do emissions scale proportionately with population, or does pollution tend to outpace or lag urbanization? (2) Are the linkages between urbanization, pollution, and economic growth inevitable, or can environmental policies decouple the seemingly structural relationship between population and pollution without affecting the link between population and economic growth?

This paper employs scaling analysis in order to explore the association between urbanization, pollution, and economic output. This scaling relationship typically assumes the following form:

$$Y_{i,t}=\beta_{0}\varepsilon_{i,t}N_{i,t}^{\alpha_{0}}$$

where (Y_i,t_) is some outcome of interest for unit (i) in time period (t), (N_i,t_) is a measure of system size, *β*_0_, *α*_0_ are statistically estimated parameters, and *ε*_*i,t*_ are unobserved factors that affect the outcome. The parameter of interest is the scaling term *α*_0_. There is a substantial body of existing research that has documented scaling relationships both in how cities are formed and in how urbanization affects various economic and environmental outcomes [3,4,5,6,7,8,9]. Most relevant to the present paper, previous studies report that carbon dioxide (CO_2_) emissions scale linearly (or even super-linearly) with city size in the United States [10,11,12,13]. Other authors find evidence of sublinear scaling between city size and satellite-based measures of ambient nitrogen dioxide [14].

Our paper makes three contributions. This is the first paper to empirically quantify how local pollution scales with population and economic output using annual data from all settlements in the United States. Second, we estimate these scaling relationships not just for physical emissions, but also for the monetary damages from these emissions. This distinction is critical, as human welfare doesn’t depend simply on the mass of emissions but rather their costs to society. Third, greenhouse gases (GHGs) are largely unregulated in the United States, while local air pollution is often tightly controlled. Exploiting heterogeneity in the regulatory treatment of local air pollution, this paper provides the first test of whether environmental policy actually reduces the scaling factor between population and pollution. We also examine whether these environmental regulations have economic costs; namely, does environmental regulation disrupt the super-linear scaling relationship between population and economic output found in previous work [3]?

To empirically test for scaling in pollution, we assemble county-level data for the United States for the following five years: 1999, 2002, 2005, 2008, and 2011. Our measures of pollution include CO_2_ emissions and five local air pollutants: fine particulate matter (PM_2.5_), sulfur dioxide (SO_2_), nitrogen oxides (NO_x_), volatile organic compounds (VOC), and ammonia (NH_3_). The CO_2_ emissions data are from [3] and the local pollution data are from the U.S. Environmental Protection Agency’s (EPA’s) National Emissions Inventory (NEI) which is released in the years listed above, [15–19]. The EPA is a federal agency whose responsibilities include measuring both global and local pollution and determining whether counties are in compliance with federal environmental regulations such as the Clean Air Act. The NEI is the most comprehensive emissions database for air pollutants. It consists of measured emissions for millions of point sources, and estimated emissions for smaller sources such as vehicles and households. While using estimated emissions is not ideal, especially given that these estimates potentially depend on population and compliance status with the Clean Air Act (among other factors). However, we emphasize that only smaller sources of emissions are estimated rather than measured, and we are not aware of any database that measures emissions from these sources at a national scale for our 1999–2011 sample period.

A new dimension of our work relative to [10,11,12,13,14] is the calculation of the monetary damage from emissions. For local pollutants, monetary damages reflect human exposures and they are calculated using an integrated assessment model [20–23]. For each source and pollutant, marginal damages are estimated in dollars per ton. These damages are multiplied by emissions to compute *Gross External Damage* (GED), [20], which is an environmental accounting analog to *Gross Domestic Product* (GDP). CO_2_ emissions are valued at the Social Cost of Carbon (SCC) [24]. Considering monetary damage rather than simply emissions is a critically important extension of prior work because monetization facilitates aggregation across different pollutants in a manner that reflects the different per-ton marginal damages of these pollutants. For example, though the mass of CO_2_ emissions dwarfs the mass of emissions from local pollutants, local pollutants are typically much more harmful per ton. The marginal damages from different types of local pollution (ex: PM_2.5_ versus NO_x_) are also not equivalent in value [25]. Summarizing, the only way to aggregate impacts from both CO_2_ emissions and local pollutants is to sum up the total damages from these different pollutants (in dollars) rather than emissions (in tons) or marginal damages (in dollars per ton).

We define settlements in two ways: metropolitan statistical areas (MSAs) and core based statistical areas (a category which includes both micropolitan and metropolitan areas) because integrated human systems do not necessarily follow county or state boundaries. MSAs are comprised of counties, and each county can only be in one MSA. Our independent variables of interest are population counts for these settlements as well as two measures of economic output: settlement-level Gross Domestic Product (GDP) and personal income [26]. A critical contribution of our paper relative to prior work is examining the role environmental policy plays in shaping the scaling relationships linking population and economic output to pollution. To explore this, the paper uses annual data provided by the EPA regarding whether each county was in compliance with the National Ambient Air Quality Standards (NAAQS) established by the Clean Air Act [27]. These ambient standards are set at the national level, and a county is deemed to be “out of attainment” if monitored pollution levels within this county are above the standard. The NAAQS are typically calibrated annually according to average or maximum pollution targets. Our measure of a county’s attainment history for a given year-of-sample is whether the county was out of attainment with the NAAQS in *any* previous year. If a county is determined to be out of attainment, state air pollution regulators typically submit a plan to achieve attainment that is reviewed by the EPA. The propensity for a county to be in non-attainment is affected by a number of factors, including industrial composition, population density, as well as local topography and climate.

We consider the following estimating equation for settlement (i) in year (t):

$$ln(Y_{i,t})=ln\beta_{0}+\alpha_{0}ln(N_{i,t})+ln(\epsilon_{i,t})$$

where *Y*_*i,t*_ is the outcome variable (either emissions, total environmental damages, marginal environmental damages, or economic output depending on specification) and *N*_*i,t*_ is our independent variable of interest (either population, gross domestic product, or personal income depending on specification). Our coefficient of interest is the scaling parameter (*α*_0_). We estimate this equation both using maximum likelihood assuming log(*ϵ*_*i,t*_) is independently and identically distributed with a Normal distribution and ordinary least squares (OLS). We use the methodology and code developed by [28] for the maximum likelihood (ML) estimation.

Both OLS and ML yield very similar point estimates. However, we reject the joint Null hypothesis that our model is correctly specified and the error terms (log(*ϵ*_*i,t*_)) are homoscedastic using the methodology described in (28). Due to the need to account for heteroscedasticity in the errors, we present OLS estimates with 95% confidence intervals around our scaling parameter estimate based on standard errors clustered by settlement; this clustering strategy allows for arbitrary correlation within settlement. Clustering results in 95% confidence intervals around our scaling parameter estimate that are conservative; using heteroscedasticity-consistent standard errors or a bootstrap procedure results in tighter 95% confidence intervals than those reported below. The error bars computed for the ML estimates using [28] are also tighter than those reported below. We present the estimates and error bars for the ML estimation procedure in the Supplementary Materials.

## Results

Table 1 displays the pooled results for settlements in the United States over the following five years: 1999, 2002, 2005, 2008, and 2011. We see from Column 2 of Table 1 that emissions scale at roughly three-quarters power of population; our estimated scaling exponents are statistically significant for both specifications using all settlements and restricting only to MSAs. Moreover, the maximum likelihood estimates for the same relationship from S2 Table are 0.71 for all settlements (0.72, for MSAs only); this similarity between OLS and ML results gives us confidence that our empirical findings are not an artifact of which estimation procedure is used. The fact that emissions of local air pollutants scale sublinearly with population stands in stark contrast to evidence indicating that CO_2_ emissions scale linearly (or even super-linearly) with population [10,11,12,13]. One candidate hypothesis for this departure from prior work is that local air pollutants are subject to various forms of environmental regulation whereas CO_2_ emissions are largely unregulated. To the authors’ knowledge, this is the first empirical evidence of three-quarters power scaling in pollution and population.

**Table 1:** Pooled scaling exponents for local air pollutants – 1999 through 2011.

		GED		Emissions		Marginal Damage	
	Definition Of Size	Exponent	R^2^ (N)	Exponent	R^2^ (N)	Exponent	R^2^ (N)
**All** **Settlements**	Population	0.95 (0.92,0.98)^A^	0.68 (4,530) ^B^	0.72 (0.69,0.75)	0.64 (4,530)	0.36 (0.31,0.40)	0.22 (4,530)
	Personal Income	0.86 (0.83,0.89)	0.65 (4,530)	0.65 (0.62,0.68)	0.62 (4,530)	0.33 (0.29,0.37)	0.22 (4,530)
**MSAs**	Population	1.03 (0.98,1.09)	0.68 (1,875)	0.75 (0.71,0.79)	0.65 (1,875)	0.33 (0.26,0.39)	0.18 (1,875)
	Personal Income	0.91 (0.85,0.96)	0.64 (1,875)	0.65 (0.61,0.69)	0.60 (1,875)	0.28 (0.23,0.34)	0.16 (1,875)
	Metro. GDP	0.89 (0.83,0.94)	0.66 (1,500)	0.63 (0.59,0.67)	0.62 (1,500)	0.25 (0.19,0.31)	0.13 (1,500)

Table 1 presents scaling parameters linking population and economic output (personal income and GDP) with local air pollution (emissions, marginal damages, and total damages) estimated using ordinary least squares.

A = 95% confidence interval based on standard errors clustered by settlement in parentheses.

B = Number of observations in parentheses.

An important implication of this finding is that, as populations continue to urbanize due to the various benefits that living in a city provides, emissions of local pollutants will not keep pace. Evaluated at the estimated coefficients reported in Table 1, a 10-fold increase in population yields just a 5.6 times increase in local emissions under the three-quarter power law. The environmental implications of sublinear scaling in emissions are massive when considering that hundreds of millions of people live in U.S. urban centers.

Our findings also imply that emissions per person scale at roughly negative one quarter power with population. Put another way, urbanization reduces per capita emissions even though aggregate emissions increase due to increased demand for goods and services. However, we are treating each ton of pollutant equally in our specifications considering emissions; we simply sum up tons of emissions of PM_2.5_, SO_2_, NO_x_, VOC, and NH_3_ to obtain our measure of local pollutant emissions. This equal weighting does not capture differences in the environmental costs from each of these pollutants.

The proper way to aggregate the impact of different pollutants is to add up the total monetary damage from each of these pollutants. This is how we calculate gross emissions damages (GED, in dollars), which is the product of emissions (in tons) and marginal damage (in dollars per ton). Column 1 of Table 1 reports evidence of near-linear scaling between GED with population. The reported exponent of 0.95 in all settlements and 1.03 in MSAs suggests that, although emissions growth is sublinear in population, the consequences of such emissions rise linearly with city size. Mathematically, this finding is due to the fact that the scaling coefficient relating population and GED is the sum of the scaling coefficients linking population to emissions and population to marginal damages (see the methods section below). Empirically, this pattern is likely because only the emissions of local pollutants are regulated; the population exposures and damages from these emissions are not directly regulated. For example, if an environmental regulation imposes a certain level of emissions, environmental damages still increase with population because more people are exposed to this level of emissions.

The findings in Table 1 are potentially troubling given that the adverse effects from pollution are ultimately what matter to human welfare rather than simply the quantity of emissions. The structure of cities reduces environmental burden per capita *in terms of emissions*. However, the finding of linear scaling means that *damages per capita* remain constant as population increases. This underscores the importance of exploring the scaling relationship between monetary damages and population as opposed to emissions and population, as the previous literature has done [10,11,12,13,14].

### Pollution and the economic output of cities

Column 2 of Table 1 indicates that a similar intuition applies when exploring how emissions scale with economic output (measured either by personal income or GDP). In particular, we estimate that emissions scale at only a 0.65 power with economic output. Thus, though economic growth spurred by urbanization will increase emissions, per-capita emissions decrease as a function of economic output.

However, total damages (GED) from pollution exposure increases only slightly less than linearly with either measure of economic output (Column 1 of Table 1) for both MSAs and all settlements. An implication of this finding is that total damages fall per unit of economic output; for example, a 10% increase in personal income induces a 1.5% reduction in damage-per unit of income. It is well known that cities generate scale economies: cost savings per unit of economic output. The results of this study suggest that cities produce social scale economies as well: lower environmental damages per unit of output. Put another way, the return to economic output (measured either by GDP or personal income) of increasing population is greater than the associated pollution costs. This is a powerful result with respect to the net benefits from further urbanization.

Agglomeration and urbanization are known to boost productivity [1,2]. Also, the authors in [3] report that measures of innovation and wealth creation increase super-linearly with population size using data from cities in United States, China and Europe. We similarly show in the Supplementary Materials that both GDP and personal income also increase more than proportionally with population in the United States from 1999–2011. In particular, the scaling exponent estimates for population and either measure of economic output are approximately 1.10. Therefore, since gross emission damage scales linearly with population and economic output scales super-linearly with population, the economic benefits from increasing population outweigh the environmental costs due to local air pollution and CO_2_ emissions.

### Inclusion of CO_2_ for 1999 through 2008

Table 2 reports the scaling coefficients combining the monetary damages from local pollutants together with CO_2_ emissions. These results do not include 2011 as the CO_2_ data do not cover this year. Table 2 shows that the scaling coefficient linking GED and population including the damages from CO_2_ is statistically indistinguishable from the coefficient estimate excluding CO_2_: both are about 0.97. Similarly, the scaling relationship between economic output (personal income and GDP) and damages including versus excluding CO_2_ are statistically indistinguishable.

**Table 2:** Pooled scaling exponents for CO_2_ and local air pollutants – 1999 through 2008.

**GED from both Local Pollutants and CO**_**2**_			
**Area**	**Definition of Size**	**Exponent** **(95% C.I.)**	**R^2^** **(N)**
**All Settlements**	Population	0.97 (0.93,1.00)^A^	0.70 (3,428)^B^
	Personal Income	0.86 (0.83,0.90)	0.66 (3,428)
**MSAs**	Population	1.03 (0.97,1.09)	0.69 (1,476)
	Personal Income	0.91 (0.85,0.96)	0.65 (1,476)
	Metro GDP	0.88 (0.83,0.94)	0.67 (1,107)
**GED from Local Pollutants**			
**Area**	**Definition of Size**	**Exponent** **(95% C.I.)**	**R^2^** **(N)**
**All Settlements**	Population	0.98 (0.94,1.01)	0.68 (3,428)
	Personal Income	0.87 (0.84,0.90)	0.64 (3,428)
**MSAs**	Population	1.05 (0.99,1.11)	0.67 (1,476)
	Personal Income	0.92 (0.87,0.98)	0.63 (1,476)
	Metro GDP	0.90 (0.85,0.96)	0.65 (1,107)

The top panel of Table 2 reports the scaling coefficient estimates linking population and economic output (personal income and GDP) with the total monetary damages from local pollutants combined with CO_2_ emissions. The bottom panel of Table 2 reports the scaling coefficient estimates linking population and economic output (personal income and GDP) with the total monetary damages from just local pollutants. These scaling coefficients are estimated using ordinary least squares.

A = 95% confidence interval based on standard errors clustered by settlement in parentheses.

B = Number of observations in parentheses.

Recall that GED is the product of marginal damages and emissions; thus, the weights applied in calculating the GED are the marginal damages per ton of each pollutant. Pollutants with higher monetary impacts per ton are attributed more weight in the GED. As is well-known, CO_2_ is a global stock pollutant with low marginal damage [24]; the per-ton damage from local pollutants can be up to several orders of magnitude larger than the marginal damage from CO_2_ [25,29,30]. Moreover, because CO_2_ is a global pollutant, the per-ton damages from CO_2_ emissions do not depend on the location of their source. Due to these factors, CO_2_ emissions have a small impact on how total environmental damages scale with population. This underscores the importance of the marginal damage weights in aggregating different pollutants.

**Fig 1:**
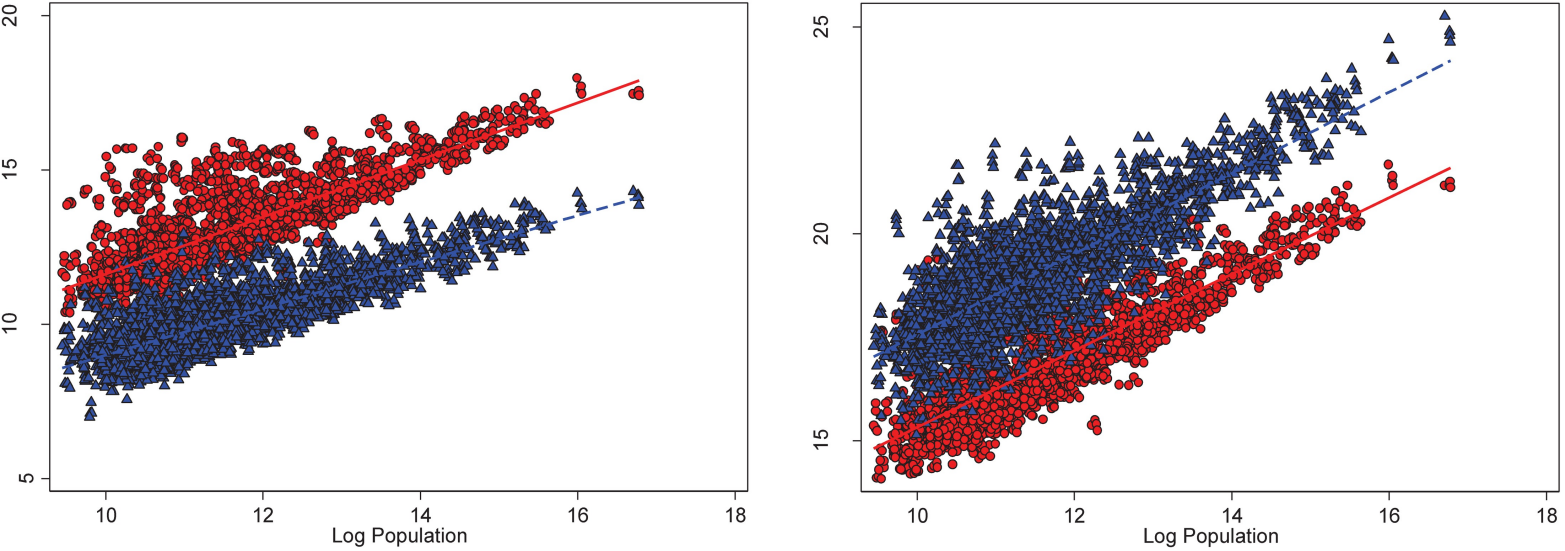
Pollution emissions and damages plotted against population. The left panel of Fig 1 shows a scatterplot of annual, settlement-level CO_2_ emissions (circles) and aggregated local pollution emissions (triangles) against population for all areas; the solid, red line is the best fit line between CO_2_ and population and the dashed, blue line is the best fit line between local air pollution and population. The right panel of Fig 1 plots annual, settlement-level gross emissions damages (GED) from CO_2_ emissions (circles) and aggregated local pollution emissions (triangles) against population; the solid, red line is the best fit line between the GED from CO_2_ emissions and population and the dashed, blue line is the best fit line between the GED from local air pollution and population.

Fig 1 illustrates why population scales differently with emissions versus total damages. Namely, the left panel of Fig 1 shows a scatterplot of annual, settlement-level CO_2_ emissions (circles) and aggregated local pollution emissions (triangles) against population for all areas; CO_2_ emissions exceed air pollution emissions in most cases. We also see that the scaling exponent for CO_2_ (the slope of the solid, red line) is greater than the slope for local air pollution (the slope of the dashed, blue line). The right panel plots annual, settlement-level GED against population; in contrast with emissions in tons, the monetary damages from local air pollution exceed the monetary damages from CO_2_ emissions in most cases. Moreover, the scaling coefficients linking population to GED are nearly identical for CO_2_ emissions (the slope of the solid, red line) versus local pollution (the slope of the dashed, blue line) because local pollutants are more harmful than CO_2_ emissions on a per-ton basis.

### Scaling and regulation

In this subsection, we test the hypothesis that environmental regulations aimed at mitigating local air pollutants contribute to the ¾ power law between local pollutants and population. The top panel of Table 3 shows the estimated power law exponents between annual-level population and pollution (emissions, marginal damages, and total damages) using only counties that *have ever been* out of attainment with the NAAQS. Counties that are, or have been, in non-attainment are subject to more stringent emission controls than compliant areas; thus, the results from Table 3 indicate how the relationship between population and pollution is affected by stricter environmental regulations.

**Table 3:** Scaling exponents for population and pollution by attainment status with the Clean Air Act.

**Counties Out of Attainment with Clean Air Act**						
	**GED**		**Emission**		**Marginal Damage**	
**Pollutant(s)**	Exponent (95% C.I.)	R^2^ (N)	Exponent (95% C.I.)	R^2^ (N)	Exponent (95% C.I.)	R^2^ (N)
**CO**_**2**_	0.58 (0.40,0.76)^A^	0.41 (248)^B^	0.58 (0.40,0.76)	0.41 (248)		
**Local** **Pollutants**	0.72 (0.54,0.89)	0.58 (248)	0.55 (0.39,0.70)	0.47 (248)	0.05 (-0.10,0.19)	0.01 (248)
**Counties In Attainment with Clean Air Act**						
	**GED**		**Emission**		**Marginal Damage**	
**Pollutant(s)**	Exponent (95% C.I.)	R^2^ (N)	Exponent (95% C.I.)	R^2^ (N)	Exponent (95% C.I.)	R^2^ (N)
**CO**_**2**_	0.93 (0.88,0.98)	0.56 (3,292)	0.93 (0.88,0.98)	0.56 (3,292)		
**Local** **Pollutants**	0.94 (0.90,0.97)	0.65 (3,292)	0.74 (0.71,0.77)	0.65 (3,292)	0.41 (0.37,0.45)	0.29 (3,292)

The top panel of Table 3 shows the estimated power law exponents between annual-level population and both local pollution as well as CO_2_ (emissions, marginal damages, and total damages) using only counties that *have ever been* out of attainment with the NAAQS. The bottom panel of Table 3 shows the estimated power law exponents between annual-level population and both local pollution as well as CO_2_ (emissions, marginal damages, and total damages) using only counties that have no history of nonattainment with the NAAQS. These scaling parameters are estimated using ordinary least squares. Coefficient estimates for CO_2_ marginal damages are excluded because these marginal damages do not differ by settlement for a given year.

A = 95% confidence interval based on standard errors clustered by settlement in parentheses.

B = Number of observations in parentheses.

The results from Table 3 indicate stark differences in the scaling relationship between population and pollution for counties always in attainment (“attainment counties”) versus counties ever out of attainment (“non-attainment counties”). Now, instead of the linear scaling reported in [10,11,12,13], CO_2_ emissions only scale to roughly the ½ power of population for non-attainment counties. Local pollutant emissions also scale sublinearly with population (with a scaling coefficient of roughly 0.55). In addition, total damages from both CO_2_ emissions and local pollutants scale sublinearly with population in non-attainment counties; our estimated coefficient is 0.58 for CO_2_ and 0.72 for local pollutants. Finally, marginal damages from local pollutants do not systematically scale with population for the subset of counties ever out of attainment with the NAAQS.

It is intuitive that the scaling coefficients are lower in non-attainment counties; policy enforcement limits emissions. In doing so, regulation breaks down the power law relationships observed in Tables 1 and 2. One explanation for this finding is that emission levels tend to be relatively higher in low population counties out of attainment. These counties typically feature large industrial point sources such as power plants. Of course, the decision to place, or maintain, power plants in lower population areas is influenced by the NAAQS; siting new large facilities is typically not permitted in counties at or near the NAAQS limits. Thus, regulatory constraints affect emission intensity at extant plants (through abatement) as well as siting decisions for new plants. Both factors likely impact the difference in scaling coefficients for counties in versus out of attainment.

The bottom panel of Table 3 reports the scaling exponents only for counties with no history of non-attainment. CO_2_ emissions scale with population according to a 0.93 power law, which is roughly in line with the literature [10,11,12,13]. Likewise, we estimate a scaling exponent of 0.75 for local air pollutants, which is similar to our findings in Table 1. Finally, as in Table 2, the scaling relationship between population and total damages for counties in attainment is close to linear for both CO_2_ and local pollutants. In the Supplementary Materials, we provide the results from regressions pooling both attainment and non-attainment counties where we statistically test whether the relationship between population and pollution varies by attainment status. The results from these pooled regressions indicate that non-attainment counties have statistically lower scaling coefficients relating population to both emissions and GED relative to counties that were always in attainment.

**Fig 2:**
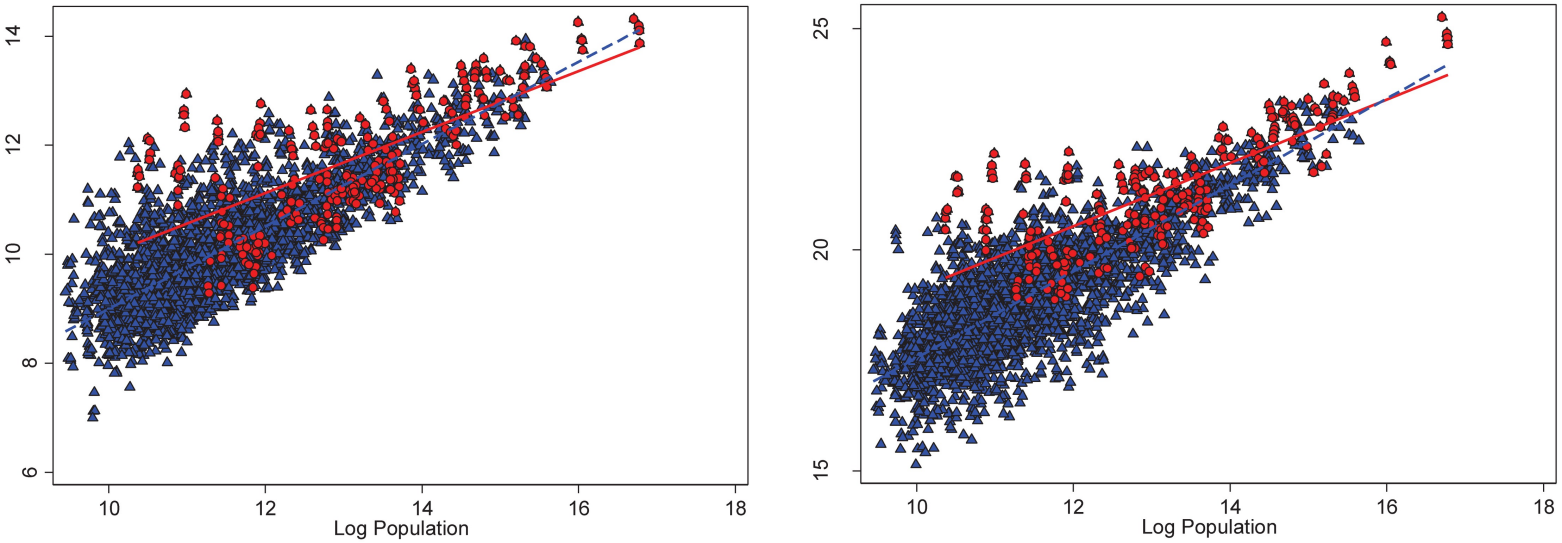
Local pollution emissions and damages plotted against population: By attainment status with the Clean Air Act. The left panel of Fig 2 plots annual, county-level local pollutant emissions against population, separately for counties that were ever out of attainment with the NAAQS (denoted by red circles and the solid, red linear fit line) and counties always in attainment with the NAAQS (denoted by blue triangles and the dashed, blue linear fit line). The right panel of Fig 2 plots annual, county-level total damages from local pollution against population, again separately for attainment versus non-attainment counties.

Fig 2 presents this intuition graphically. In particular, the left panel of Fig 2 plots annual, county-level local pollutant emissions against population, separately for counties that were ever out of attainment with the NAAQS (denoted by red circles and the solid, red linear fit line) and counties always in attainment with the NAAQS (denoted by blue triangles and the dashed, blue linear fit line). We see from this figure that: 1) there are far fewer county-year observations in the “non-attainment” category relative to the “attainment” category, and 2) population scales with local emissions at a lower power for non-attainment counties relative to attainment counties. The right panel of Fig 2 plots annual, county-level total damages from local pollution against population, again separately for attainment versus non-attainment counties. As with emissions, we see that the total damages from local pollutants scale at a lower power for counties that have ever been out of attainment with NAAQS relative to counties that have always been in attainment with NAAQS.

With respect to continued urbanization, enforcement of the NAAQS provides clear benefits in reducing per capita emissions and environmental damages. What are the economic costs of these policies? Broadly, the costs of environmental policy are comprised of foregone opportunities; production or consumption of goods and services are curtailed in order to reduce emissions and comply with the NAAQS. Bearing this in mind, a regression of annual, settlement-level economic output (personal income and metropolitan GDP) on population *in non-attainment counties* serves as a simple test of whether enforcement of the NAAQS breaks down the super-linear relationship between population and economic output observed in previous work [3]. In the Supplementary Materials, we present the scaling coefficients for GDP and personal income against population for all counties versus only for non-attainment counties. The results from this statistical test show conclusively that GDP and personal income increase super-linearly (or at least linearly) with population even in non-attainment counties; there is no evidence that a county being in non-attainment adversely affects the returns to agglomeration. Summarizing, we present evidence that environmental regulations such as the NAAQS can result in sub-linear scaling between population and environmental costs without disrupting the super-linear scaling between population and economic output. This mitigates concerns that environmental policy must inevitably slow economic growth in order to reduce emissions.

## Discussion

Firms locate in cities to exploit productivity gains. The global population is urbanizing in pursuit of a higher quality of life. Aside from offering economies of scale for firms and employment opportunities, cities tend to economize on energy use and the delivery of basic services through infrastructure. Many of these benefits from urbanization can be succinctly expressed through scaling laws that depict the percent change in a quantity of interest relative to population size [3,4,5,6,8]; this raises the question of whether the waste generated in cities also scales systematically with city size. Most of the prior work in this field finds linear (or even super-linear) scaling between CO_2_ emissions and population [10,11,12,13]. The implications of this scaling relationship between population and pollution are significant: will environmental outcomes improve or worsen as the global population continues to urbanize?

We move the literature forward by expanding the scope of pollution considered, by modeling both pollution emissions and monetary damages, and by exploring whether scaling laws vary as a function of environmental policy. Our paper has three central findings. First, emissions of local air pollutants increase according to a three quarters power law in population size; thus, local emissions per capita decrease with population. Second, we estimate a roughly linear relationship between the total monetary damages from emissions and population whether we consider the combined total damages from CO_2_ and local air pollution or just local pollution damages. Finally, our analysis shows that environmental damages scale with population at a much smaller power for counties that faced stricter environmental regulation due to previously being out of attainment with the Clean Air Act. In contrast, the scaling law between population and economic output is similar for counties in versus out of attainment.

Counties in non-attainment are typically subject to more rigorous emission controls. For non-attainment (attainment) counties, the 0.55 (0.74) power law for emissions and population as well as the 0.72 (0.94) power law for total damages and population suggest that environmental policy effectively mitigates the extent to which emissions and damages scale with population. However, opponents of environmental policy often emphasize that such laws are costly; they argue that environmental controls inhibit production and impede growth. We present statistical evidence against this argument; we find for U.S. counties between 1999 and 2011 that both metropolitan GDP and personal income scale super-linearly with population size regardless of whether we consider counties in versus out of attainment with the NAAQS. Therefore, it appears that policy can dramatically reduce the adverse environmental consequences of urbanization while not stifling society’s furnaces of production, innovation, and growth.

As discussed above, urbanization impacts a variety of different city characteristics, including productivity, innovation, road networks, congestion, and industrial structure [1,2,3,4,5,8,9]. It is important to note that this paper does not disentangle the channels through which population and economic output affect pollution; we simply estimate the scaling relationship between population, economic output and pollution inclusive of all of these channels. Separately identifying different channels linking population to pollution, which we leave to future work, might allow policymakers to target environmental regulations to the city characteristics that most affect pollution while least affecting economic output.

## Methods

### Calculation of marginal damages

This subsection provides a detailed discussion of how we use the AP2 Integrated Assessment Model (IAM) to calculate marginal damages; AP2 is an updated version of an IAM used in prior publications [20–23, 31]. This model links the emissions of five common air pollutants to estimates of ambient concentrations, exposures, physical health environmental impacts, and monetary damage. This paper considers the following local air pollutants: nitrogen oxides (NO_x_), sulfur dioxide (SO_2_), fine particulate matter (PM_2.5_), ammonia (NH_3_), and volatile organic compounds (VOC). The data years (1999, 2002, 2005, 2008, and 2011) comprise the years for which National Emissions Inventories (NEI) are published by the EPA, [15–19]. These data include all reported emissions of the five pollutants listed above for each data year in the contiguous United States.

We use the EPA emissions data combined with an air quality model to link emissions to annual average concentrations by county. The validity of AP2’s predicted air quality conditions is tested in [31]; the model satisfies most performance evaluation standards. The model calculates exposures by tracking predicted concentrations and populations of sensitive receptors (human populations and crops, for example) at the county level for each year in the analysis. Physical impacts (reduced crop yields, increased mortality rates, among others) are estimated using concentration-response functions from peer-reviewed publications. The functions that connect exposure to elevated mortality risk are the most important in determining the level of monetary damage from local air pollutants since the largest share of damages from these pollutants come from these increases in mortality risk [32]. This analysis employs the results from [33] for exposure to PM_2.5_; [33] is a recent update to the most commonly used concentration-response function [32]. AP2 employs the results from [34] for tropospheric O_3_.

We place a monetary value on these impacts from emissions using either reported market prices (for crops) or standard non-market valuation techniques. Importantly, AP2 uses the Value of a Statistical Life (VSL) approach to monetize increases in mortality [35]; in particular, this study uses the EPA’s preferred VSL of $6 million (in year 2000 dollars) [32]. We should note that our mortality-based approach may under-state the total costs of local air pollution; in particular, local air pollution has been linked with increased hospitalizations due to respiratory illness such as asthma, increased prevalence of crime, as well as a decrease in the value of urban assets [36,37,38].

The AP2 model computes source-and-pollutant-specific marginal damages for each of the five air pollutants in five different data years; these marginal damages are estimated using the algorithm developed in [25]. For a given year, this algorithm attributes emissions of each pollutant to one of 10,000 sources in AP2 according to the EPA’s NEI [15–19]. Next, AP2 is run in order to estimate total monetary damages at baseline emission levels. Then, one ton of one pollutant is added to the baseline emissions for the first source in the NEI; the model is then run again. AP2 computes the change (with respect to the baseline) in concentrations, exposures, physical effects, and damages due to including this additional ton of pollutant. The difference between baseline damages and damages under the add-one-ton scenario is the monetary damage from the additional ton. This algorithm calculates the spatial sum (across counties that are affected by the emitted pollution) of the difference in damages as:

$$MD_{jts}=\sum_{r=1}^{R}\left(D^{a}{}_{rts}-D^{b}{}_{rts}\right)$$

where: D = monetary damage in receptor county (r), time (t), species (s)

a = add-one-ton emission scenario
b = baseline emission scenario

The algorithm is repeated for all five pollutants, for the 10,000 sources, and the five data years for a total of 250,000 marginal damage estimates. The marginal damages are then matched to emissions data by source (j), pollutant (s), and year (t) to compute GED:

$$GED_{t}=\sum_{s}\sum_{j}MD_{j,t,s}\times E_{j,t,s}$$

CO_2_ emissions data are provided by [3]. We use a social cost of CO_2_ emissions of $40/ton CO_2_, which is the value calculated in [24]; total CO_2_ damages are computed by multiplying reported emissions by this per-ton social cost of CO_2_. Thus, both local air pollutants and CO_2_ are valued through a price-times-quantity metric.

### Data and estimation

Power laws typically assume the form shown in Equation (1) below; in our context, the population (or economic output) in settlement (i) at time (t) is given by (*N*_*i*,*t*_) and a measure of pollution (either emissions or damages) is given by (Y_*i*,*t*_).

$$Y_{i,t}=\beta_{0}\varepsilon_{i,t}N_{i,t}^{\alpha_{0}}$$

where the error term *ε*_*i,t*_ consists of other factors that affect the outcome *Y*_*i,t*_. The parameters {*β*_0_,*α*_0_} are estimated using both maximum likelihood (ML) and ordinary least squares (OLS) on the log-transformed variables, {log(*Y*_*i,t*_),log(*N*_*i,t*_)}. We use population data provided by the U.S. Census Bureau’s intercensal county population estimates for each data year covered in the analysis (1999, 2002, 2005, 2008, and 2011). We explore two additional specifications based on economic output, measured by settlement-level GDP and personal income. "Settlements" in our analysis can consist either of metropolitan statistical areas (MSAs) or core based statistical areas (a category which includes both micropolitan and metropolitan areas). As discussed above, we consider three types of pollution measurements: emissions, marginal (or per-ton) damages, and total damages (GED).

Defining emissions in terms of population using the functional form in Equation (1) gives us: $E_{i,t}=\beta_{e}\epsilon_{i,t}{\left(N_{i,t}\right)}^{\alpha_{e}}$; we can similarly specify marginal damages as: ${MD}_{i,t}=\beta_{m}u_{i,t}{\left(N_{i,t}\right)}^{\alpha_{m}}$. Combining these two equations imply that GED can be re-written as: ${GED}_{i,t}={\beta_{e}\beta }_{m}\epsilon_{i,t}u_{i,t}{\left(N_{i,t}\right)}^{\left(\alpha_{m}+\alpha_{e}\right)}$. Thus, how *GED* scales with population depends on the sum of the scaling exponents for emissions and marginal damages.

## Supporting information

S1 Fig Pollution emissions and damages per-capita plotted against population. The left panel of S1 Fig plots local air pollution emissions divided by population against population. The right panel of S1 Fig shows the total damages from these emissions (GED) divided by population against population. The solid, red line is fit to non-attainment counties, with red circles denoting each county/year observation associated with non-attainment counties. County/year observations for attainment counties are represented by blue triangles, along with a dashed, blue linear regression fit line for these observations.
(TIF)

S1 Table Scaling exponents for local air pollutants, changes over time– 1999 through 2011. S1 Table presents regression results allowing our scaling exponents relating local air pollution (emissions, marginal damages, and total damages) to population and economic output (personal income and GDP) to vary by year-of-sample.
(DOCX)

S2 Table Pooled scaling exponents for local air pollutants– 1999 through 2011 (log-normal maximum likelihood estimates). S2 Table presents scaling parameters linking population and economic output (personal income and GDP) with local air pollution (emissions, marginal damages, and total damages) estimated using maximum likelihood.
(DOCX)

S3 Table Pooled scaling exponents for CO_2_ and local air pollutants– 1999 through 2008: Log-normal MLE. S3 Table presents scaling parameters linking population and economic output (personal income and GDP) with the combined total damages from local pollution and CO_2_ emissions estimated using maximum likelihood.
(DOCX)

S4 Table Scaling exponents for population and attainment with the Clean Air Act: Log-normal MLE. S4 Table presents scaling parameters linking population with local pollution and CO_2_ (emissions, marginal damages, and total damages) estimated separately for counties in versus out of attainment with the NAAQS using maximum likelihood.
(DOCX)

S5 Table Economic output and population size: Log-normal MLE. S5 Table presents scaling parameters linking population with economic output (personal income and GDP) estimated separately for counties in versus out of attainment with the NAAQS using maximum likelihood.
(DOCX)

S6 Table Economic output and population size. S6 Table presents scaling parameters linking population with economic output (personal income and GDP) estimated separately for counties in versus out of attainment with the NAAQS using ordinary least squares.
(DOCX)

S7 Table Relative scaling exponents for population and non-attainment with the Clean Air Act. S7 Table presents estimates of the change in the scaling parameter linking population with local pollution and CO_2_ (emissions, marginal damages, and total damages) for counties that are out of attainment with the NAAQS relative to in attainment with the NAAQS. These parameters are estimated using ordinary least squares.
(DOCX)

S1 Data Data files used in this analysis. This zip file contains all of the raw data files used in this analysis.
(ZIP)

S1 Text Additional description of supplementary materials. This Word document provides additional details regarding the supplementary figures and tables described above.
(DOCX)
