# Supplementary figures and images for: Does environmental policy affect scaling laws between population and pollution? Evidence from American metropolitan areas
Source: PLoS One. 2017 Aug 9;12(8):e0181407. doi: 10.1371/journal.pone.0181407 (PMC5549900; doi:10.1371/journal.pone.0181407)

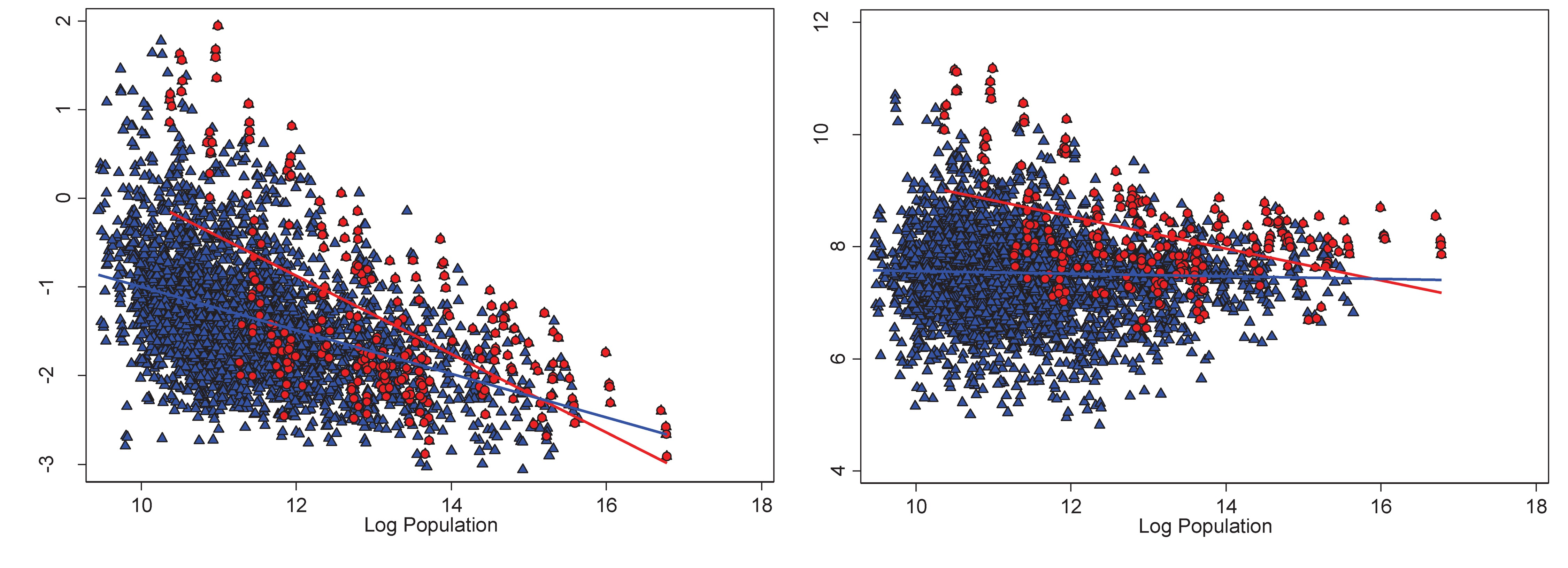

Supplement: S1 Fig — The left panel of S1 Fig plots local air pollution emissions divided by population against population. The right panel of S1 Fig shows the total damages from these emissions (GED) divided by population against population. The solid, red line is fit to non-attainment counties, with red circles denoting each county/year observation associated with non-attainment counties. County/year observations for attainment counties are represented by blue triangles, along with a dashed, blue linear regression fit line for these observations. (TIF) [file pone.0181407.s001.tif]
