# Supplementary material for: Does environmental policy affect scaling laws between population and pollution? Evidence from American metropolitan areas
Source: PLoS One. 2017 Aug 9;12(8):e0181407. doi: 10.1371/journal.pone.0181407 (PMC5549900; doi:10.1371/journal.pone.0181407)
Supplement: S1 Table — presents regression results allowing our scaling exponents relating local air pollution (emissions, marginal damages, and total damages) to population and economic output (personal income and GDP) to vary by year-of-sample. (DOCX) [file pone.0181407.s002.docx]

S1 Table: Scaling Exponents for Local Air Pollutants, Changes over Time – 1999 through 2011

|  | Gross External  Damage | | Emissions | | Marginal  Damage | |
| --- | --- | --- | --- | --- | --- | --- |
| Population | Exponent | Adj. R^2^ | Exponent | Adj. R^2^ | Exponent | Adj. R^2^ |
| 1999 | 0.98  (0.94,1.02)^A,B^ | 0.72 | 0.77  (0.74,0.80) | 0.70 | 0.41  (0.37,0.45) | 0.32 |
| 2002 | 0.981  (0.94,1.02) | 0.70 | 0.78  (0.75,0.81) | 0.72 | 0.42  (0.39,0.46) | 0.34 |
| 2005 | 0.969  (0.93,1.01) | 0.72 | 0.76  (0.72,0.79) | 0.69 | 0.40  (0.36,0.44) | 0.31 |
| 2008 | 1.02  (0.97,1.06) | 0.70 | 0.74  (0.71,0.78) | 0.67 | 0.37  (0.33,0.41) | 0.26 |
| 2011 | 0.82  (0.79,0.86) | 0.70 | 0.57  (0.54,0.60) | 0.57 | 0.17  (0.12,0.21) | 0.06 |
| Personal  Income | Exponent | Adj. R^2^ | Exponent | Adj. R^2^ | Exponent | Adj. R^2^ |
| 1999 | 0.91  (0.87,0.94) | 0.71 | 0.70  (0.67,0.74) | 0.68 | 0.37  (0.34,0.41) | 0.30 |
| 2002 | 0.90  (0.87,0.94) | 0.70 | 0.71  (0.68,0.74) | 0.71 | 0.39  (0.35,0.43) | 0.34 |
| 2005 | 0.89  (0.85,0.93) | 0.71 | 0.70  (0.67,0.73) | 0.68 | 0.37  (0.33,0.41) | 0.30 |
| 2008 | 0.94  (0.90,0.98) | 0.68 | 0.69  (0.66,0.72) | 0.67 | 0.35  (0.31,0.38) | 0.26 |
| 2011 | 0.77  (0.73,0.80) | 0.68 | 0.53  (0.50,0.56) | 0.55 | 0.14  (0.10,0.18) | 0.05 |
| GDP | Exponent | Adj. R^2^ | Exponent | Adj. R^2^ | Exponent | Adj. R^2^ |
| 2002 | 0.92  (0.86,0.98)^C^ | 0.69 | 0.68  (0.63,0.72) | 0.68 | 0.29  (0.23,0.35) | 0.21 |
| 2005 | 0.91  (0.84,0.97) | 0.68 | 0.67  (0.62,0.72) | 0.66 | 0.29  (0.23,0.35) | 0.18 |
| 2008 | 0.95  (0.88,1.01) | 0.67 | 0.68  (0.63,0.73) | 0.66 | 0.29  (0.23,0.35) | 0.19 |
| 2011 | 0.83  (0.77,0.88) | 0.71 | 0.55  (0.50,0.60) | 0.58 | 0.11  (0.04,0.17) | 0.02 |

S1 Table presents regression results allowing our scaling exponents relating local air pollution (emissions, marginal damages, and total damages) to population and economic output (personal income and GDP) to vary by year-of-sample.

A = 95% confidence intervals in parentheses.

B = 906 observations.

C = 375 observations.
