# Supplementary material for: Does environmental policy affect scaling laws between population and pollution? Evidence from American metropolitan areas
Source: PLoS One. 2017 Aug 9;12(8):e0181407. doi: 10.1371/journal.pone.0181407 (PMC5549900; doi:10.1371/journal.pone.0181407)
Supplement: S2 Table — presents scaling parameters linking population and economic output (personal income and GDP) with local air pollution (emissions, marginal damages, and total damages) estimated using maximum likelihood. (DOCX) [file pone.0181407.s003.docx]

S2 Table: Pooled Scaling Exponents for Local Air Pollutants – 1999 through 2011 (Log-Normal Maximum Likelihood Estimates)

|  | GED | | Emissions | | | Marginal Damages | | | |
| --- | --- | --- | --- | --- | --- | --- | --- | --- | --- |
|  | Exponent  (95% C.I.) | NLL | Exponent  (95% C.I.) | NLL | | Exponent  (95% C.I.) | NLL | |  |
| Definition  Of Size | **All Settlements** | | | | | | | | |
| Personal  Income | 0.85  (0.83,0.87)^A^ | 91,948.8 | 0.65  (0.63,0.67) | | 50,879.3 | 0.31  (0.28,0.34) | | 45,038.0 | |
| Population | 0.93  (0.91,0.95) | 91,724.0 | 0.71  (0.69,0.73) | | 50,711.8 | 0.33  (0.30,0.36) | | 45,042.2 | |
|  | **MSAs** | | | | | | | | |
| Population | 1.00  (0.97,1.03) | 39,706.2 | 0.72  (0.70,0.74) | | 22,232.9 | 0.30  (0.25,0.35) | | 19,252.2 | |
| Personal  Income | 0.88  (0.85,0.91) | 39,829.6 | 0.63  (0.61,0.65) | | 22,388.0 | 0.26  (0.22,0.30) | | 19,267.3 | |
| Metro.  GDP | 0.86  (0.83,0.89) | 31,713.8 | 0.61  (0.58,0.64) | | 17,775.5 | 0.22  (0.18,0.26) | | 15,416.6 | |

S2 Table presents scaling parameters linking population and economic output (personal income and GDP) with local air pollution (emissions, marginal damages, and total damages) estimated using maximum likelihood.

A = 95% confidence interval based on the bootstrap procedure in parentheses.
