# Supplementary material for: Does environmental policy affect scaling laws between population and pollution? Evidence from American metropolitan areas
Source: PLoS One. 2017 Aug 9;12(8):e0181407. doi: 10.1371/journal.pone.0181407 (PMC5549900; doi:10.1371/journal.pone.0181407)
Supplement: S3 Table — presents scaling parameters linking population and economic output (personal income and GDP) with the combined total damages from local pollution and CO2 emissions estimated using maximum likelihood. (DOCX) [file pone.0181407.s004.docx]

S3 Table: Pooled Scaling Exponents for CO_2_ and Local Air Pollutants – 1999 through 2008: Log-Normal MLE

| **GED from both Local Pollutants and CO_2_** | | | |
| --- | --- | --- | --- |
| **Area** | **Definition of Size** | **Exponent**  **(95% C.I.)** | **NLL** |
| **All** **Settlements** | Population | 0.95  (0.93,0.97)^A^ | 69,581.2 |
|  | Personal Income | 0.85  (0.83,0.89) | 69,524.7 |
| **MSAs** | Population | 1.00  (0.97,1.03) | 31,330.4 |
|  | Personal Income | 0.88  (0.85,0.91) | 31,435.1 |
|  | Metro GDP | 0.85  (0.82,0.88) | 23,450.1 |
| **GED from Local Pollutants** | | | |
| **Area** | **Definition of Size** | **Exponent**  **(95% C.I.)** | **NLL** |
| **All** **Settlements** | Population | 0.95  (0.93,0.97) | 69,311.9 |
|  | Personal Income | 0.85  (0.83,0.87) | 69,798.3 |
| **MSAs** | Population | 1.01  (0.98,1.04) | 31,229.5 |
|  | Personal Income | 0.89  (0.86,0.92) | 31,330.2 |
|  | Metro GDP | 0.87  (0.84,0.90) | 23,365.8 |

S3 Table presents scaling parameters linking population and economic output (personal income and GDP) with the combined total damages from local pollution and CO_2_ emissions estimated using maximum likelihood.

A = 95% confidence interval based on the bootstrap procedure in parentheses.
