# Supplementary material for: Does environmental policy affect scaling laws between population and pollution? Evidence from American metropolitan areas
Source: PLoS One. 2017 Aug 9;12(8):e0181407. doi: 10.1371/journal.pone.0181407 (PMC5549900; doi:10.1371/journal.pone.0181407)
Supplement: S4 Table — presents scaling parameters linking population with local pollution and CO2 (emissions, marginal damages, and total damages) estimated separately for counties in versus out of attainment with the NAAQS using maximum likelihood. (DOCX) [file pone.0181407.s005.docx]

S4 Table: Scaling Exponents for Population and Attainment with the Clean Air Act: Log-Normal MLE

| Counties Out of Attainment with Clean Air Act | | | | | | |
| --- | --- | --- | --- | --- | --- | --- |
|  | **GED** | | **Emissions** | | **Marginal Damage** | |
| Pollutant(s) | Exponent  (95% C.I.) | NLL | Exponent  (95% C.I.) | NLL | Exponent  (95% C.I.) | NLL |
| CO_2_ | 0.66  (0.59,0.73)^A^ | 4,930.7 | 0.64 (0.55,0.73) | 4,015.4 | 0.10  (0.04,0.16) | 2,741.1 |
| Local  Pollutants | 0.79  (0.69,0.89) | 5,556.1 | 0.59 (0.53,0.65) | 3,156.3 | 0.10  (0.04,0.16) | 2,741.1 |
| Counties In Attainment with Clean Air Act | | | | | | |
|  | **GED** | | **Emissions** | | **Marginal Damage** | |
| Pollutant(s) | Exponent  (95% C.I.) | NLL | Exponent  (95% C.I.) | NLL | Exponent  (95% C.I.) | NLL |
| CO_2_ | 0.86  (0.82,0.90) | 59,113.3 | 0.86  (0.82,0.90) | 46,969.5 | 0.39  (0.36,0.42) | 31,346.0 |
|  |  |  |  |  |  |  |
| Local  Pollutants | 0.91  (0.89,0.93) | 65,885.6 | 0.72  (0.70,0.74) | 35,905.1 | 0.39  (0.36,0.42) | 31,346.0 |
|  |  |  |  |  |  |  |

S4 Table presents scaling parameters linking population with local pollution and CO_2_ (emissions, marginal damages, and total damages) estimated separately for counties in versus out of attainment with the NAAQS using maximum likelihood.

A = 95% confidence interval based on the bootstrap procedure in parentheses.
