# Supplementary material for: Does environmental policy affect scaling laws between population and pollution? Evidence from American metropolitan areas
Source: PLoS One. 2017 Aug 9;12(8):e0181407. doi: 10.1371/journal.pone.0181407 (PMC5549900; doi:10.1371/journal.pone.0181407)
Supplement: S5 Table — presents scaling parameters linking population with economic output (personal income and GDP) estimated separately for counties in versus out of attainment with the NAAQS using maximum likelihood. (DOCX) [file pone.0181407.s006.docx]

S5 Table: Economic Output and Population Size: Log-Normal MLE

|  | Metro.  GDP | | Personal  Income | |
| --- | --- | --- | --- | --- |
| Population | Exponent  (95% C.I.) | NLL  (N) | Exponent  (95% C.I.) | NLL  (N) |
| All  Settlements | 1.12  (1.11,1.13)^A^ | 14,210.0  (1,500)^B^ | 1.07  (1.06,1.08) | 53,501.1  (3,624) |
| Non-  Attainment | 1.09  (1.05,1.13) | 2,329.0  (220) | 1.10  (1.08,1.12) | 4,289.5  (264) |
| Attainment | 0.96  (0.92,1.00) | 14,494.9  (1,329) | 1.06  (1.05,1.07) | 50,915.1  (3,476) |

S5 Table presents scaling parameters linking population with economic output (personal income and GDP) estimated separately for counties in versus out of attainment with the NAAQS using maximum likelihood.

A = 95% confidence interval based on the bootstrap procedure in parentheses.

B = Number of observations in parentheses.
