# Supplementary material for: Does environmental policy affect scaling laws between population and pollution? Evidence from American metropolitan areas
Source: PLoS One. 2017 Aug 9;12(8):e0181407. doi: 10.1371/journal.pone.0181407 (PMC5549900; doi:10.1371/journal.pone.0181407)
Supplement: S6 Table — presents scaling parameters linking population with economic output (personal income and GDP) estimated separately for counties in versus out of attainment with the NAAQS using ordinary least squares. (DOCX) [file pone.0181407.s007.docx]

S6 Table: Economic Output and Population Size.

|  | Metro.  GDP | | Personal  Income | |
| --- | --- | --- | --- | --- |
| Population | Exponent  (95% C.I.) | R^2^  (N) | Exponent  (95% C.I.) | R^2^  (N) |
| All  Settlements | 1.11  (1.08,1.14)^A^ | 0.94  (1,500)^B^ | 1.065  (1.055,1.074) | 0.98  (3,624) |
| Non-  Attainment | 1.12  (1.05,1.19) | 0.94  (220) | 1.09  (1.07,1.11) | 0.99  (264) |
| Attainment | 0.94  (0.84,1.04) | 0.58  (1,392) | 1.06  (1.05,1.07) | 0.97  (3,476) |

S6 Table presents scaling parameters linking population with economic output (personal income and GDP) estimated separately for counties in versus out of attainment with the NAAQS using ordinary least squares.

A = 95% confidence interval based on the bootstrap procedure in parentheses.

B = Number of observations in parentheses.
