# Supplementary material for: Does environmental policy affect scaling laws between population and pollution? Evidence from American metropolitan areas
Source: PLoS One. 2017 Aug 9;12(8):e0181407. doi: 10.1371/journal.pone.0181407 (PMC5549900; doi:10.1371/journal.pone.0181407)
Supplement: S7 Table — presents estimates of the change in the scaling parameter linking population with local pollution and CO2 (emissions, marginal damages, and total damages) for counties that are out of attainment with the NAAQS relative to in attainment with the NAAQS. These parameters are estimated using ordinary least squares. (DOCX) [file pone.0181407.s008.docx]

S7 Table: Relative Scaling Exponents for Population and Non-Attainment with the Clean Air Act.

|  | Regression with Counties Both In and Out of Attainment with Clean Air Act | | | | | |
| --- | --- | --- | --- | --- | --- | --- |
|  | **GED** | | **Emissions** | | **Marginal Damage** | |
| Pollutant(s) | Exponent  (95% C.I.) | R^2^ | Exponent  (95% C.I.) | R^2^ | Exponent  (95% C.I.) | R^2^ |
| CO_2_ | -0.23  (-0.40,-0.06)^A,B^ | 0.70 | -0.35  (-0.52,-0.18) | 0.61 | * | * |
| Local  Pollutants | -0.22  (-0.39,-0.05) | 0.71 | -0.19  (-0.35,-0.04) | 0.69 | -0.36  (-0.50,-0.22) | 0.39 |

S7 Table presents estimates of the change in the scaling parameter linking population with local pollution and CO_2_ (emissions, marginal damages, and total damages) for counties that are out of attainment with the NAAQS relative to in attainment with the NAAQS. These parameters are estimated using ordinary least squares. We report the coefficient estimate (with 95% C.I.s) of the interaction between log of population and an indicator which is one if the county has even been out of attainment and zero otherwise. Marginal damages from CO_2_ emissions do not vary across counties because CO_2_ is a global pollutant; this is why we do not estimate the scaling relationship between marginal damages and population when considering only CO_2_ emissions.

A = 95% confidence interval based on standard errors clustered by settlement in parentheses.

B = All of these regressions are based on N= 3,540 observations.
