# Supplementary material for: Does environmental policy affect scaling laws between population and pollution? Evidence from American metropolitan areas
Source: PLoS One. 2017 Aug 9;12(8):e0181407. doi: 10.1371/journal.pone.0181407 (PMC5549900; doi:10.1371/journal.pone.0181407)
Supplement: S1 Text — This Word document provides additional details regarding the supplementary figures and tables described above. (DOCX) [file pone.0181407.s010.docx]

**Additional Description of Supplementary Materials**

C**hanges in the scaling exponents over time**

We allow our scaling exponents relating local air pollution to population and economic output to vary by year-of-sample in S1 Table. The dominant pattern in all three cases (emissions, marginal damages, and total damages) when examining the results where population is the independent variable is that the scaling exponent falls in 2011 relative to the other data years. Namely, emissions scale with population according to a three-quarter power law from 1999 to 2008. However, the exponent falls to under 0.60 in 2011. Similarly, marginal damages scale at slightly larger than 1/3^rd^ power with population from 1999 to 2008; this scaling exponent falls to about 1/6^th^ in 2011. Finally, GED scales linearly with population from 1999 to 2008, but at only the 0.82 power of population in 2011.

S1 Table: Scaling Exponents for Local Air Pollutants, Changes over Time – 1999 through 2011

|  | Gross External  Damage | | Emissions | | Marginal  Damage | |
| --- | --- | --- | --- | --- | --- | --- |
| Population | Exponent | Adj. R^2^ | Exponent | Adj. R^2^ | Exponent | Adj. R^2^ |
| 1999 | 0.98  (0.94,1.02)^A,B^ | 0.72 | 0.77  (0.74,0.80) | 0.70 | 0.41  (0.37,0.45) | 0.32 |
| 2002 | 0.981  (0.94,1.02) | 0.70 | 0.78  (0.75,0.81) | 0.72 | 0.42  (0.39,0.46) | 0.34 |
| 2005 | 0.969  (0.93,1.01) | 0.72 | 0.76  (0.72,0.79) | 0.69 | 0.40  (0.36,0.44) | 0.31 |
| 2008 | 1.02  (0.97,1.06) | 0.70 | 0.74  (0.71,0.78) | 0.67 | 0.37  (0.33,0.41) | 0.26 |
| 2011 | 0.82  (0.79,0.86) | 0.70 | 0.57  (0.54,0.60) | 0.57 | 0.17  (0.12,0.21) | 0.06 |
| Personal  Income | Exponent | Adj. R^2^ | Exponent | Adj. R^2^ | Exponent | Adj. R^2^ |
| 1999 | 0.91  (0.87,0.94) | 0.71 | 0.70  (0.67,0.74) | 0.68 | 0.37  (0.34,0.41) | 0.30 |
| 2002 | 0.90  (0.87,0.94) | 0.70 | 0.71  (0.68,0.74) | 0.71 | 0.39  (0.35,0.43) | 0.34 |
| 2005 | 0.89  (0.85,0.93) | 0.71 | 0.70  (0.67,0.73) | 0.68 | 0.37  (0.33,0.41) | 0.30 |
| 2008 | 0.94  (0.90,0.98) | 0.68 | 0.69  (0.66,0.72) | 0.67 | 0.35  (0.31,0.38) | 0.26 |
| 2011 | 0.77  (0.73,0.80) | 0.68 | 0.53  (0.50,0.56) | 0.55 | 0.14  (0.10,0.18) | 0.05 |
| GDP | Exponent | Adj. R^2^ | Exponent | Adj. R^2^ | Exponent | Adj. R^2^ |
| 2002 | 0.92  (0.86,0.98)^C^ | 0.69 | 0.68  (0.63,0.72) | 0.68 | 0.29  (0.23,0.35) | 0.21 |
| 2005 | 0.91  (0.84,0.97) | 0.68 | 0.67  (0.62,0.72) | 0.66 | 0.29  (0.23,0.35) | 0.18 |
| 2008 | 0.95  (0.88,1.01) | 0.67 | 0.68  (0.63,0.73) | 0.66 | 0.29  (0.23,0.35) | 0.19 |
| 2011 | 0.83  (0.77,0.88) | 0.71 | 0.55  (0.50,0.60) | 0.58 | 0.11  (0.04,0.17) | 0.02 |

S1 Table presents regression results allowing our scaling exponents relating local air pollution (emissions, marginal damages, and total damages) to population and economic output (personal income and GDP) to vary by year-of-sample.

A = 95% confidence intervals in parentheses.

B = 906 observations.

C = 375 observations.

The pattern of smaller scaling exponents in 2011 also manifests for personal income. Marginal damages scale with personal income according to a 1/3^rd^ power law from 1999 to 2008; this exponent falls to approximately 1/6^th^ in 2011. Emissions increase with personal income at roughly a 2/3^rds^ power from 1999 to 2008; in 2011, the scaling exponent falls to 0.5. The GED-income scaling coefficient lies between 0.90 and 0.95 for all years except 2011, when the GED-income relationship instead obeys a 3/4^th^ power law. A very similar pattern emerges when metropolitan GDP is used as the measure of economic output.

So what causes this change in scaling relationships in 2011? While a thorough exploration of this question is beyond the scope of our paper, one obvious candidate explanation is the financial crisis and subsequent recession that affected both gross output and the composition of the U.S. economy. Namely, the manufacturing and construction sectors were hit particularly hard by the financial crisis. These sectors both have a significant effect on metropolitan economies; manufacturing facilities tend to be in or near cities and the construction of housing is obviously tied to cities. These sectors also contribute a non-negligible share of pollution emissions and monetary damages from these emissions [20,21].

**Scaling Parameter Estimates using Maximum Likelihood**

This section presents the scaling parameters estimated using the maximum likelihood (ML) methodology described in [28]. We obtain very similar coefficient estimates using ML as presented in all three of the tables in the main text (which present estimates using ordinary least squares). The error bars displayed in S2-S5 Tables do not represent 95% confidence intervals because we fail to reject the joint Null hypothesis that the model is correctly specified and the errors are independently and identically distributed using the statistical procedure described in [28]. It is for this reason that we choose to feature the OLS-based results with standard errors clustered at the settlement level in the main text instead of these ML-based results. The ML estimates have tighter (less conservative) 95% confidence intervals relative to those presented in the main text.

It is important to note that we are not trying to maximize fit when estimating these models; there will inevitably be other variables that can be added to the scaling model that improve fit. The crucial assumption underlying our estimation strategy is that the scaling relationships between population, pollution, and economic output are not instead capturing other unobserved variables correlated with both the independent and dependent variables, which would change the interpretation of our findings.

S2 Table: Pooled Scaling Exponents for Local Air Pollutants – 1999 through 2011 (Log-Normal Maximum Likelihood Estimates)

|  | GED | | Emissions | | | Marginal Damages | | | |
| --- | --- | --- | --- | --- | --- | --- | --- | --- | --- |
|  | Exponent  (95% C.I.) | NLL | Exponent  (95% C.I.) | NLL | | Exponent  (95% C.I.) | NLL | |  |
| Definition  Of Size | **All Settlements** | | | | | | | | |
| Personal  Income | 0.85  (0.83,0.87)^A^ | 91,948.8 | 0.65  (0.63,0.67) | | 50,879.3 | 0.31  (0.28,0.34) | | 45,038.0 | |
| Population | 0.93  (0.91,0.95) | 91,724.0 | 0.71  (0.69,0.73) | | 50,711.8 | 0.33  (0.30,0.36) | | 45,042.2 | |
|  | **MSAs** | | | | | | | | |
| Population | 1.00  (0.97,1.03) | 39,706.2 | 0.72  (0.70,0.74) | | 22,232.9 | 0.30  (0.25,0.35) | | 19,252.2 | |
| Personal  Income | 0.88  (0.85,0.91) | 39,829.6 | 0.63  (0.61,0.65) | | 22,388.0 | 0.26  (0.22,0.30) | | 19,267.3 | |
| Metro.  GDP | 0.86  (0.83,0.89) | 31,713.8 | 0.61  (0.58,0.64) | | 17,775.5 | 0.22  (0.18,0.26) | | 15,416.6 | |

S2 Table presents scaling parameters linking population and economic output (personal income and GDP) with local air pollution (emissions, marginal damages, and total damages) estimated using maximum likelihood.

A = 95% confidence interval based on the bootstrap procedure in parentheses.

S3 Table: Pooled Scaling Exponents for CO_2_ and Local Air Pollutants – 1999 through 2008: Log-Normal MLE

| **GED from both Local Pollutants and CO_2_** | | | |
| --- | --- | --- | --- |
| **Area** | **Definition of Size** | **Exponent**  **(95% C.I.)** | **NLL** |
| **All** **Settlements** | Population | 0.95  (0.93,0.97)^A^ | 69,581.2 |
|  | Personal Income | 0.85  (0.83,0.89) | 69,524.7 |
| **MSAs** | Population | 1.00  (0.97,1.03) | 31,330.4 |
|  | Personal Income | 0.88  (0.85,0.91) | 31,435.1 |
|  | Metro GDP | 0.85  (0.82,0.88) | 23,450.1 |
| **GED from Local Pollutants** | | | |
| **Area** | **Definition of Size** | **Exponent**  **(95% C.I.)** | **NLL** |
| **All** **Settlements** | Population | 0.95  (0.93,0.97) | 69,311.9 |
|  | Personal Income | 0.85  (0.83,0.87) | 69,798.3 |
| **MSAs** | Population | 1.01  (0.98,1.04) | 31,229.5 |
|  | Personal Income | 0.89  (0.86,0.92) | 31,330.2 |
|  | Metro GDP | 0.87  (0.84,0.90) | 23,365.8 |

S3 Table presents scaling parameters linking population and economic output (personal income and GDP) with the combined total damages from local pollution and CO_2_ emissions estimated using maximum likelihood.

A = 95% confidence interval based on the bootstrap procedure in parentheses.

S4 Table: Scaling Exponents for Population and Attainment with the Clean Air Act: Log-Normal MLE

| Counties Out of Attainment with Clean Air Act | | | | | | |
| --- | --- | --- | --- | --- | --- | --- |
|  | **GED** | | **Emissions** | | **Marginal Damage** | |
| Pollutant(s) | Exponent  (95% C.I.) | NLL | Exponent  (95% C.I.) | NLL | Exponent  (95% C.I.) | NLL |
| CO_2_ | 0.66  (0.59,0.73)^A^ | 4,930.7 | 0.64 (0.55,0.73) | 4,015.4 | 0.10  (0.04,0.16) | 2,741.1 |
| Local  Pollutants | 0.79  (0.69,0.89) | 5,556.1 | 0.59 (0.53,0.65) | 3,156.3 | 0.10  (0.04,0.16) | 2,741.1 |
| Counties In Attainment with Clean Air Act | | | | | | |
|  | **GED** | | **Emissions** | | **Marginal Damage** | |
| Pollutant(s) | Exponent  (95% C.I.) | NLL | Exponent  (95% C.I.) | NLL | Exponent  (95% C.I.) | NLL |
| CO_2_ | 0.86  (0.82,0.90) | 59,113.3 | 0.86  (0.82,0.90) | 46,969.5 | 0.39  (0.36,0.42) | 31,346.0 |
|  |  |  |  |  |  |  |
| Local  Pollutants | 0.91  (0.89,0.93) | 65,885.6 | 0.72  (0.70,0.74) | 35,905.1 | 0.39  (0.36,0.42) | 31,346.0 |
|  |  |  |  |  |  |  |

S4 Table presents scaling parameters linking population with local pollution and CO_2_ (emissions, marginal damages, and total damages) estimated separately for counties in versus out of attainment with the NAAQS using maximum likelihood.

A = 95% confidence interval based on the bootstrap procedure in parentheses.

S5 Table: Economic Output and Population Size: Log-Normal MLE

|  | Metro.  GDP | | Personal  Income | |
| --- | --- | --- | --- | --- |
| Population | Exponent  (95% C.I.) | NLL  (N) | Exponent  (95% C.I.) | NLL  (N) |
| All  Settlements | 1.12  (1.11,1.13)^A^ | 14,210.0  (1,500)^B^ | 1.07  (1.06,1.08) | 53,501.1  (3,624) |
| Non-  Attainment | 1.09  (1.05,1.13) | 2,329.0  (220) | 1.10  (1.08,1.12) | 4,289.5  (264) |
| Attainment | 0.96  (0.92,1.00) | 14,494.9  (1,329) | 1.06  (1.05,1.07) | 50,915.1  (3,476) |

S5 Table presents scaling parameters linking population with economic output (personal income and GDP) estimated separately for counties in versus out of attainment with the NAAQS using maximum likelihood.

A = 95% confidence interval based on the bootstrap procedure in parentheses.

B = Number of observations in parentheses.

**Additional Results Regarding Scaling Laws and Environmental Regulation**

**Measures of output and city size**

S6 Table displays the regression results describing how population affects measures of economic output in settlements; theses regressions use annual, MSA-level data. The top row shows that when the log of metropolitan GDP is regressed on the log of population, the scaling exponent is 1.11. Thus, economic output scales super-linearly with population. This result is very much in agreement with results from previous literature [3]. An alternative measure of economic output, gross personal income, also scales super-linearly with population; in this case, the fitted exponent is 1.07.

Considering that being in non-attainment with the NAAQS often implies tighter, more restrictive limits on the emissions of pollution (which suggests higher operating costs for firms), non-attainment with the NAAQS may potentially disrupt the super-linear relationship between population and output. However, the second row in S6 Table indicates that the super-linear relationship between population and economic output still holds for counties with a history of non-attainment with the NAAQS. We conclude from these results that facing the stricter environmental regulation that comes with non-attainment does not substantively affect how economic output changes with city size. Finally, our results are very similar if we instead estimate the scaling relationship between economic output and population using maximum likelihood instead of ordinary least squares (see S5 Table above).

S6 Table: Economic Output and Population Size.

|  | Metro.  GDP | | Personal  Income | |
| --- | --- | --- | --- | --- |
| Population | Exponent  (95% C.I.) | R^2^  (N) | Exponent  (95% C.I.) | R^2^  (N) |
| All  Settlements | 1.11  (1.08,1.14)^A^ | 0.94  (1,500)^B^ | 1.065  (1.055,1.074) | 0.98  (3,624) |
| Non-  Attainment | 1.12  (1.05,1.19) | 0.94  (220) | 1.09  (1.07,1.11) | 0.99  (264) |
| Attainment | 0.94  (0.84,1.04) | 0.58  (1,392) | 1.06  (1.05,1.07) | 0.97  (3,476) |

S6 Table presents scaling parameters linking population with economic output (personal income and GDP) estimated separately for counties in versus out of attainment with the NAAQS using ordinary least squares.

A = 95% confidence interval based on the bootstrap procedure in parentheses.

B = Number of observations in parentheses.

**How do Emissions per Capita and GED per Capita Scale with Population?**

The left panel of S1 Fig plots local air pollution emissions divided by population against population. The solid, red line is fit to non-attainment counties, with red circles denoting each county/year observation associated with non-attainment counties. County/year observations for attainment counties are represented by blue triangles, along with a dashed, blue linear regression fit line for these observations. Per-capita emissions clearly decrease less rapidly in attainment counties relative to non-attainment counties. The right panel shows the total damages from these emissions (GED) divided by population against population. As before, non-attainment counties exhibit a lower scaling factor relating per-capita GED and population relative to counties always in attainment.

**S1 Fig. Pollution Emissions and Damages Per-Capita Plotted Against Population.** The left panel of S1 Fig plots local air pollution emissions divided by population against population. The right panel of S1 Fig shows the total damages from these emissions (GED) divided by population against population. The solid, red line is fit to non-attainment counties, with red circles denoting each county/year observation associated with non-attainment counties. County/year observations for attainment counties are represented by blue triangles, along with a dashed, blue linear regression fit line for these observations.

**Direct Statistical Test of Interaction Between Attainment Status and Scaling**

In Table 3 from the main text, we present results comparing the scaling relationship between population and pollution (either emissions, total damages, or marginal damages) for counties with a history of non-attainment with the NAAQS (“non-attainment counties”) versus counties consistently in attainment with the NAAQS (“attainment counties”). This section of the Supplementary Materials provides a direct statistical test of the Null hypothesis that these scaling relationships do not vary by attainment status. In particular, we estimate the following regression equation on the pooled sample of all counties (both attainment and non-attainment) using OLS:

$$\log\left( Y_{i,t} \right)=\beta_{0}+\beta_{1}1\left( NonAttain \right)_{i}+\beta_{2}\log\left( Pop_{i,t} \right)+\beta_{3}1\left( NonAttain \right)_{i}\times\log\left( Pop_{i,t} \right)+log(\epsilon{}_{i,t})$$

where $1\left( NonAttain \right)_{i}$ is an indicator which takes the value one for non-attainment counties and the value zero otherwise and $Y_{i,t}$ is one of our three dependent variables (emissions, GED, or marginal damages). Our coefficient of interest is$\beta_{3}$; if $\beta_{3}=0$, then there is no *difference* in the scaling relationship between population and pollution for counties in versus out of attainment. S7 Table presents our coefficients estimates for the interaction term ($\hat{\beta_{3}}$) from this regression equation; we run separate specifications for each of our three pollution measures (emissions, marginal damage, and total damage) for local pollutants as well as CO_2_ emissions. We do not estimate a scaling coefficient relating population and the marginal damages from CO_2_ because CO_2_ is a global pollutant; the marginal damages from CO_2_ emissions do not vary across counties. We see from all five specifications that the interaction term between non-attainment status and population is negative and statistically different from zero. This provides compelling statistical evidence that the scaling relationship between population and pollution (both for local pollution and CO_2_) is smaller for non-attainment counties relative to attainment counties.

S7 Table: Relative Scaling Exponents for Population and Non-Attainment with the Clean Air Act.

|  | Regression with Counties Both In and Out of Attainment with Clean Air Act | | | | | |
| --- | --- | --- | --- | --- | --- | --- |
|  | **GED** | | **Emissions** | | **Marginal Damage** | |
| Pollutant(s) | Exponent  (95% C.I.) | R^2^ | Exponent  (95% C.I.) | R^2^ | Exponent  (95% C.I.) | R^2^ |
| CO_2_ | -0.23  (-0.40,-0.06)^A,B^ | 0.70 | -0.35  (-0.52,-0.18) | 0.61 | * | * |
| Local  Pollutants | -0.22  (-0.39,-0.05) | 0.71 | -0.19  (-0.35,-0.04) | 0.69 | -0.36  (-0.50,-0.22) | 0.39 |

S7 Table presents estimates of the change in the scaling parameter linking population with local pollution and CO_2_ (emissions, marginal damages, and total damages) for counties that are out of attainment with the NAAQS relative to in attainment with the NAAQS. These parameters are estimated using ordinary least squares. We report the coefficient estimate (with 95% C.I.s) of the interaction between log of population and an indicator which is one if the county has even been out of attainment and zero otherwise. Marginal damages from CO_2_ emissions do not vary across counties because CO_2_ is a global pollutant; this is why we do not estimate the scaling relationship between marginal damages and population when considering only CO_2_ emissions.

A = 95% confidence interval based on standard errors clustered by settlement in parentheses.

B = All of these regressions are based on N= 3,540 observations.
